# Supplementary material for: A distinct isoform of ZNF207 controls self-renewal and pluripotency of human embryonic stem cells
Source: Nat Commun. 2018 Oct 22;9:4384. doi: 10.1038/s41467-018-06908-5 (PMC6197280; doi:10.1038/s41467-018-06908-5)
Supplement: Supplementary file 1 — Supplementary information file [file 41467_2018_6908_MOESM1_ESM.pdf]

A distinct isoform of ZNF207 controls self-renewal and pluripotency of human embryonic stem cells

Fang et al.,

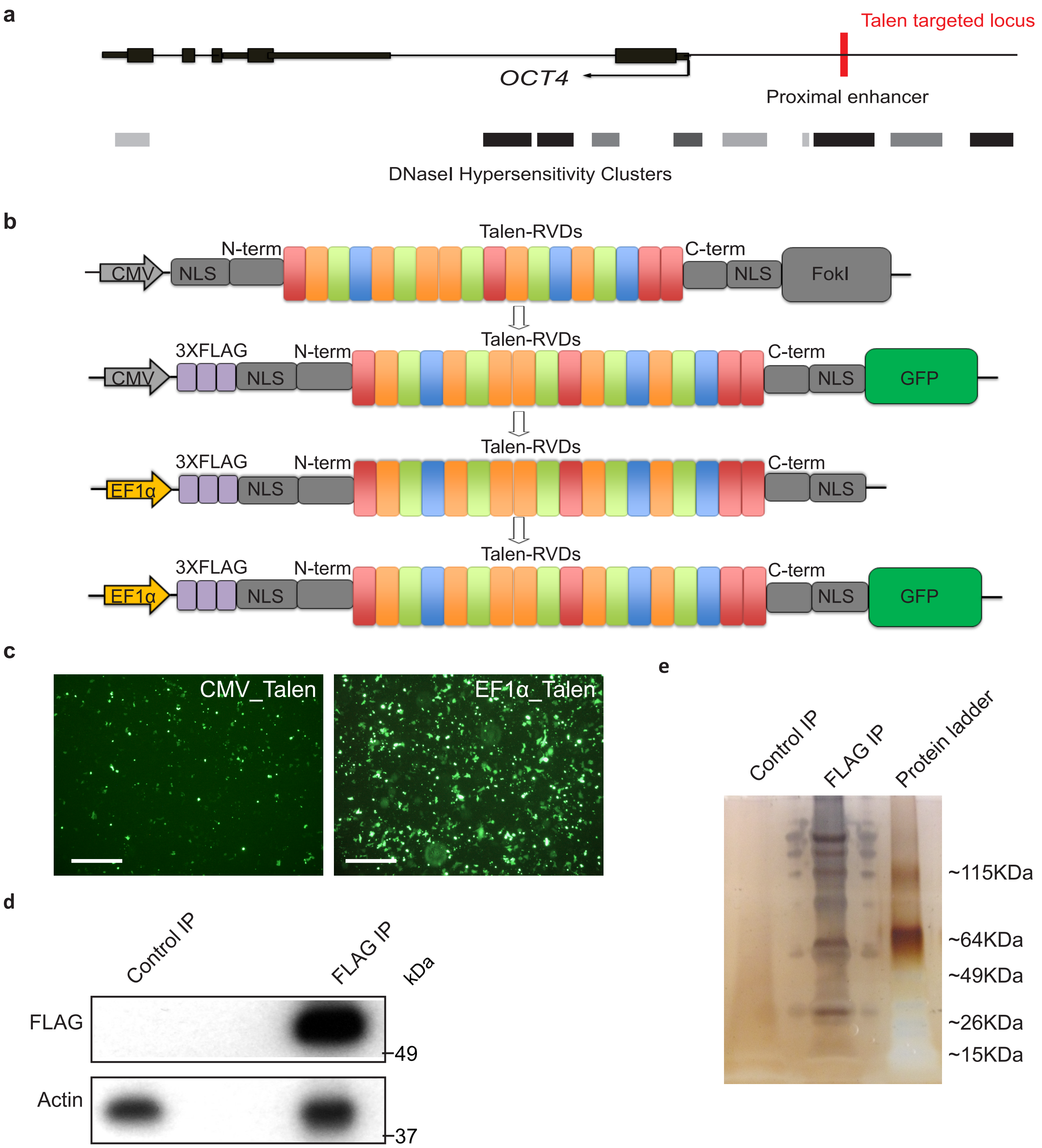

Supplementary Figure 1. Optimization of TALEN protein expression to target OCT4 locus for locus-specific proteomics in hESCs. (**a**) Schematic representation of the human OCT4 locus. Dark boxes represent exons and the red bar represents the locus targeted by TALEN protein. The extent of DNaseI hypersensitivity are indicated by gray boxes. The darkness is proportional to the maximum signal strength observed. (**b**) Schematic representation of dTALE protein designed for locus-specific proteomics in hESCs. (**c**) A comparison of transfection efficiency of CMV and EF1α promoter-driven dTALE protein in hESCs. GFP-positive cells were shown. Scale bars represent 100μm.
(**d**) Co-immunoprecipitation using nuclear extract of hESCs was performed using anti-FLAG antibody. Western blot was performed with anti-FLAG and anti-Actin antibody. Control IP was performed using an anti-green fluorescent protein (GFP) antibody. (**e**) Silver staining of the nuclear extracts of hESCs after FLAG immunoprecipitation.

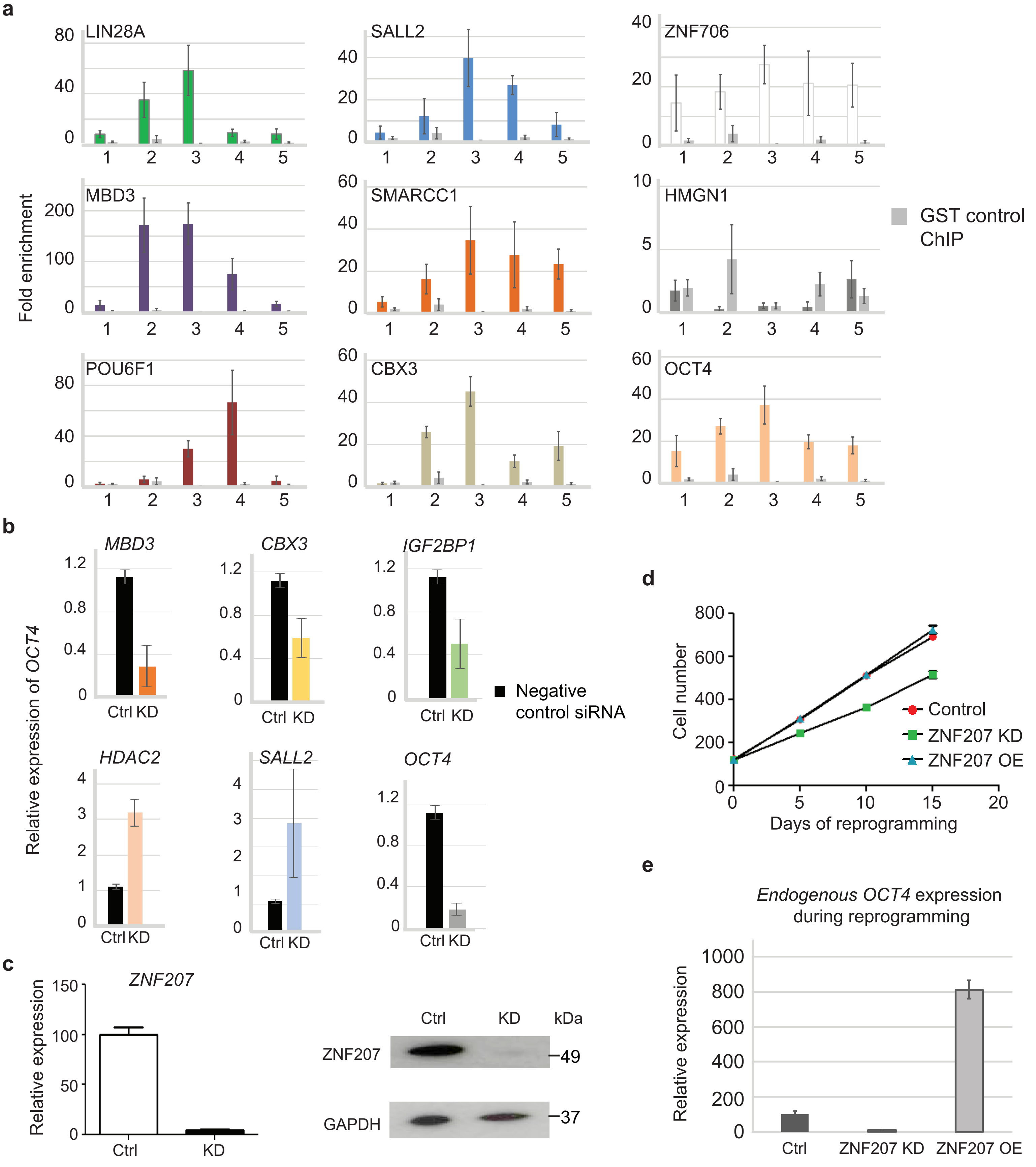

Supplementary Figure 2. Validation and functional study of a selected set of proteins identified by locus-specific proteomics at OCT4 proximal promoter. (a) ChIP assays were performed using 9 different antibodies and a control GST antibody to detect enriched fragments. Different transcription factors tested are color-coded and the labeling of the amplified products are the same as Fig. 1b. (b) Functional study of identified proteins by siRNA-mediated knockdown analysis. Quantitative RT-PCR analysis of OCT4 expression after siRNA knockdown targeting different genes. The levels of the transcripts were normalized against negative control siRNA. (c) qPCR and western blot analysis of ZNF207 expression in control and siRNA knockdown cells. (d) The counts of cell numbers during reprogramming in control, ZNF207 KD and ZNF207 OE conditions. (e) qPCR to test the endogenous OCT4 expression during reprogramming. Data are presented as the mean ±SEM and are derived from three independent experiments.

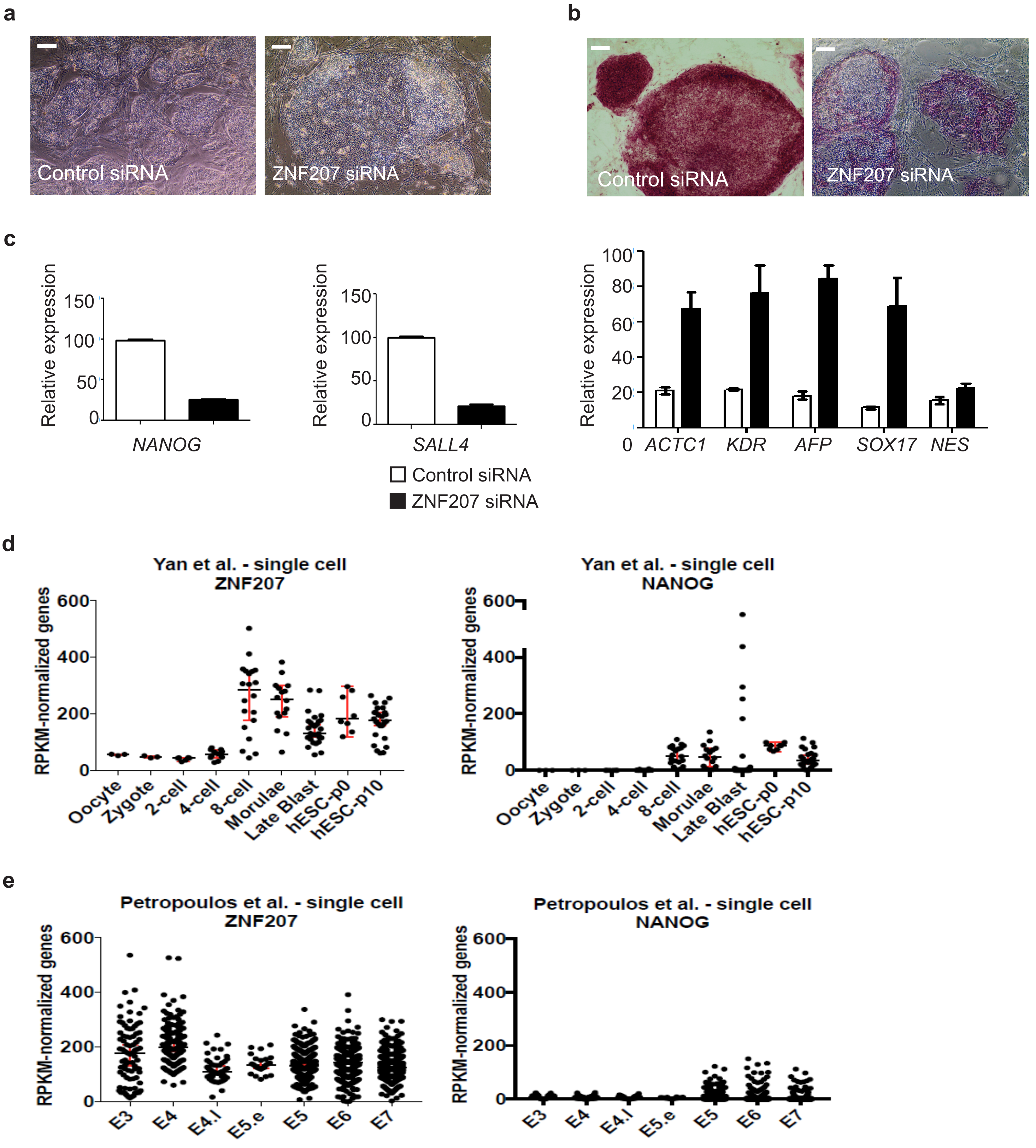

Supplementary Figure 3. Functional analysis of ZNF207 in primed and naïve pluripotent stem cells and its expression analysis during early human embryo development. (**a**) Bright field view of cell morphology after knockdown. Scale bars represent 50 µm. (**b**) Alkaline phosphatase staining of cells after knockdown. Scale bars represent 50 µm. (**c**) qPCR analysis of the expression of pluripotency and lineage marker genes in different conditions. Data are presented as the mean ±SEM and are derived from three independent experiments for qPCR analysis (**d**) The expression of ZNF207 (a) and NANOG (b) during early human embryo development based on single cell RNA-seq data generated in Yan et al., (**e**) The expression of ZNF207 and NANOG during early human embryo development based on single cell RNA-seq data generated in Petropoulos et al.,.

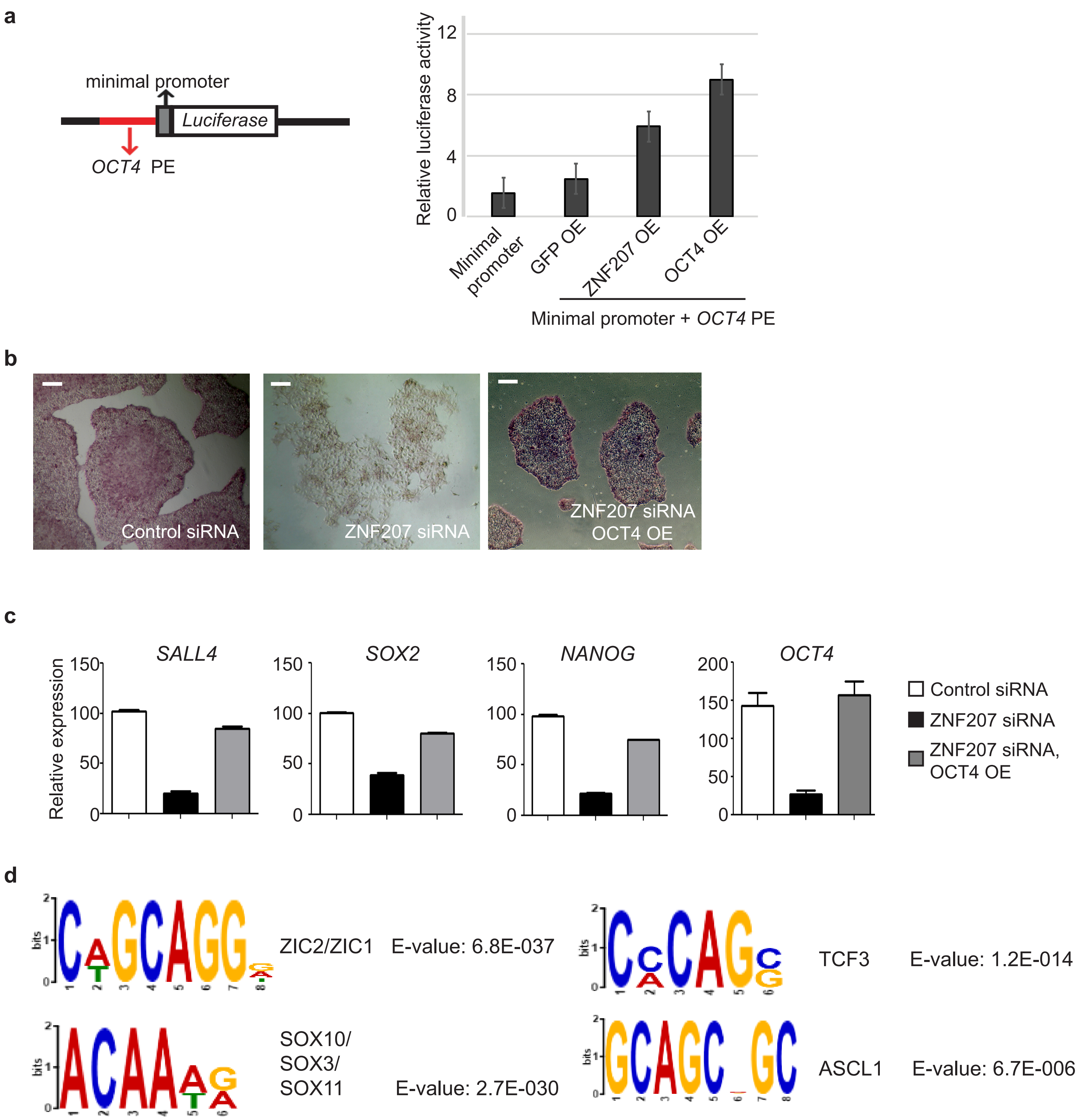

Supplementary Figure 4. Identification of direct targets of ZNF207 in hESCs. (**a**) enhancer activity is shown. Genomic fragment bound by ZNF207 (in red) was inserted upstream of a luciferase gene driven by minimal promoter. Y-axis represents the fold enrichment of luciferase activity, calculated relative to a luciferase reporter containing the minimal promoter only and normalized over an internal transfection control. (**b**) Alkaline phosphatase staining of cells after knockdown. Scale bars represent 50 μm. (**c**) qPCR analysis of the expression of pluripotency marker genes in different conditions. (**d**) Enriched motifs from de novo motif search of sequences under ZNF207 peaks. The identification of consensus binding motifs for neuronal transcription factors. Statistical significance (E-values) is indicated below the motif logo. Data are presented as the mean ±SEM and are derived from three independent experiments for qPCR analysis.

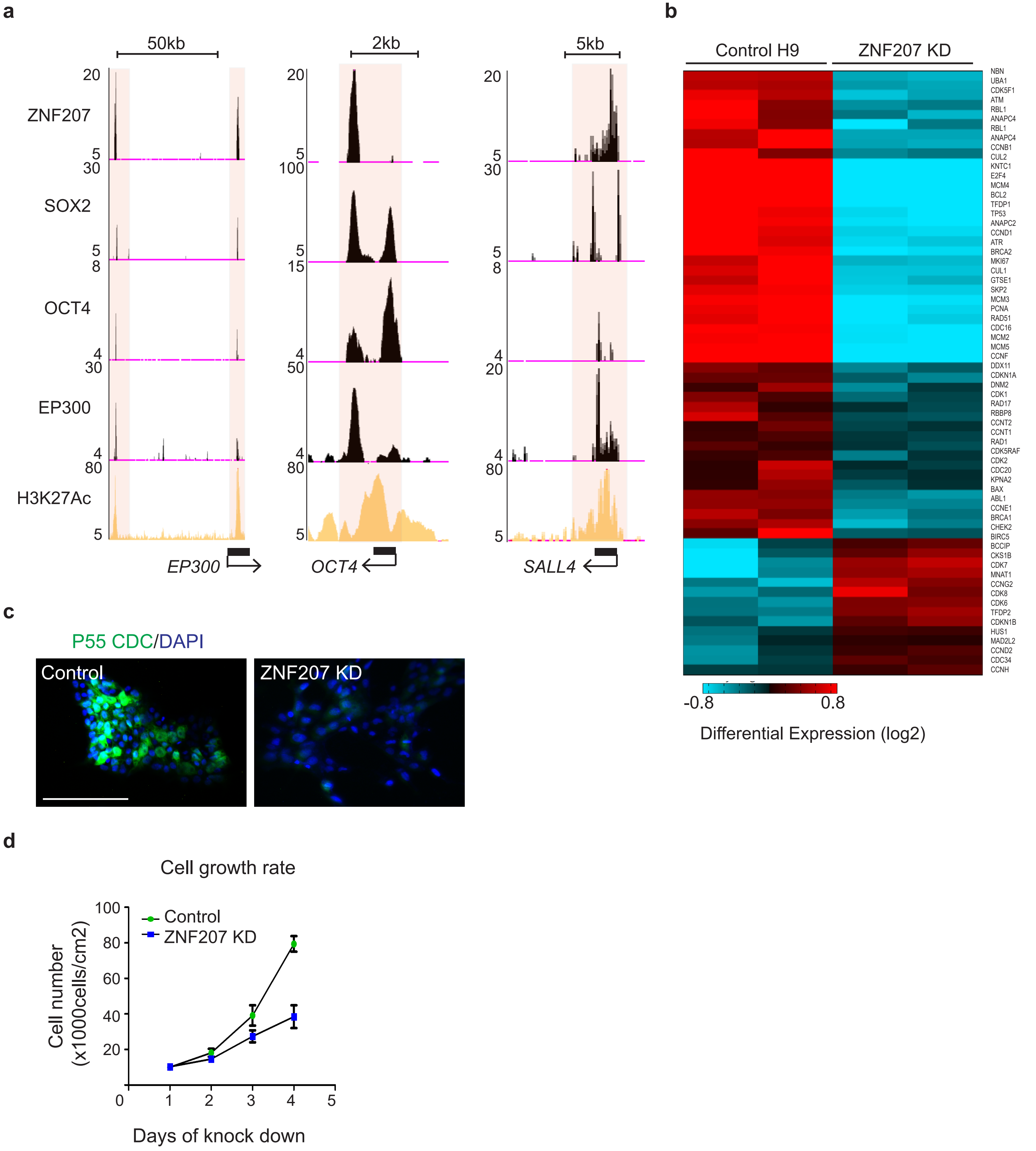

Supplementary Figure 5. ZNF207 promotes self-renewal of hESCs. (**a**) ChIP-Seq tracks show co-localization of ZNF207, SOX2, OCT4, P300 and H3K27Ac at the regulatory sequences of pluripotency genes. The scale bars indicate the size of the chromosome. The light pink boxes highlight the co-bound regions. (**b**) Heatmap showing differentially expressed cell cycle genes between control and ZNF207 KD cells; data represent our biological replicates of the same cell lines. (**c**) Immunofluorescence staining of control and ZNF207 KD hESCs for cell cycle proteins. (**d**) Comparison of cell number between ZNF207 KD and control hESCs. Data are presented as the mean ±SEM and are derived from three independent experiments.

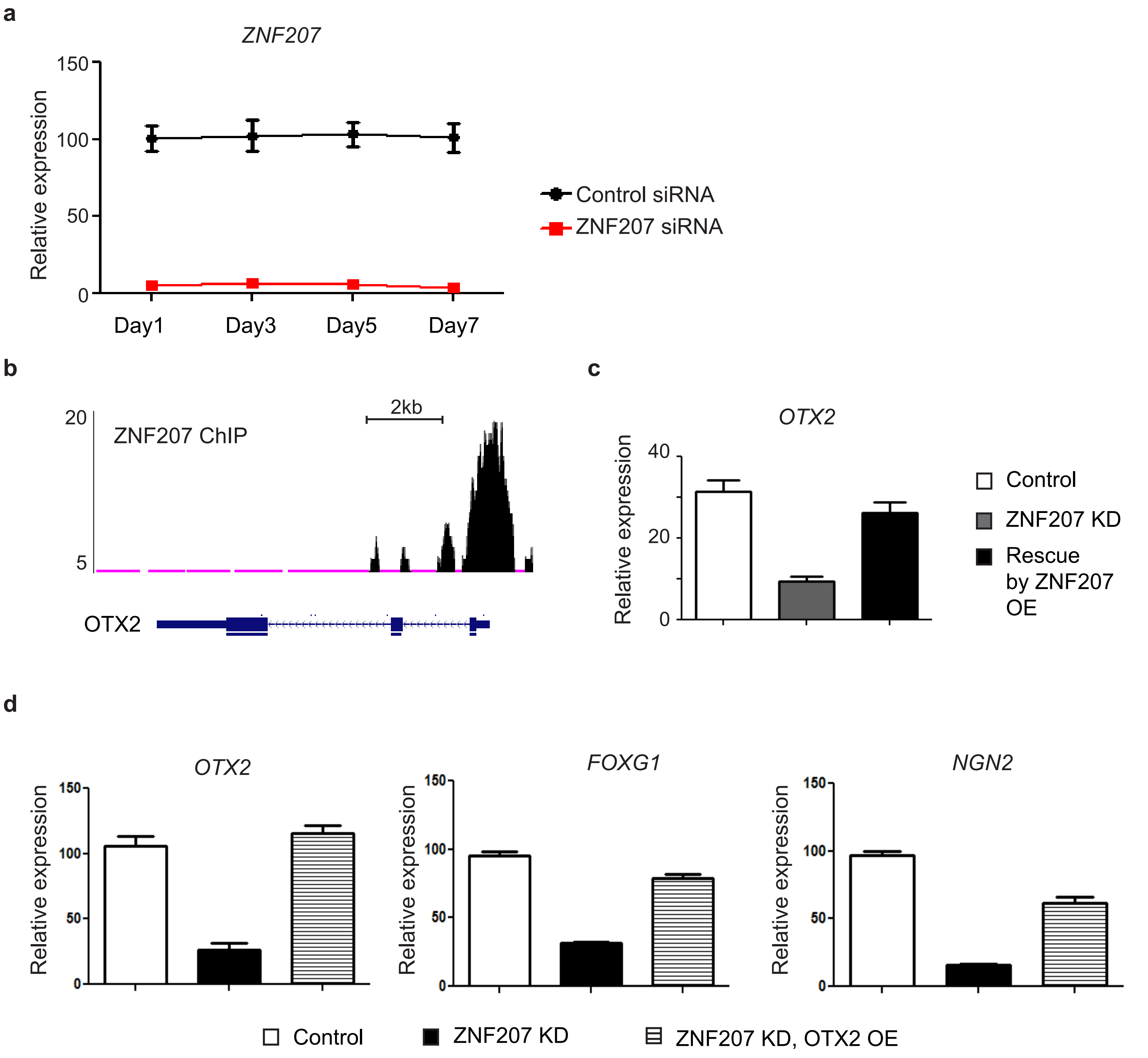

Supplementary Figure 6. ZNF207 works upstream of OTX2 to control ectoderm specification. (**a**) The expression of ZNF207 in control and ZNF207 KD cells during 7 days of differentiation. Data are presented as the mean ±SEM and are derived from three independent experiments. (**b**) ChIP-Seq tracks show binding of ZNF207 at the regulatory sequences of OTX2 gene. The scale bar indicates the size of the chromosome. (**c**) qPCR to test the expression level of OTX2 in control, ZNF207 KD and rescue by ZNF207 OE cells in the process of ectoderm differentiation. (**d**) The expression of OTX2, FOXG1 and NGN2 in different conditions. Data are presented as the mean ±SEM and are derived from three independent experiments.

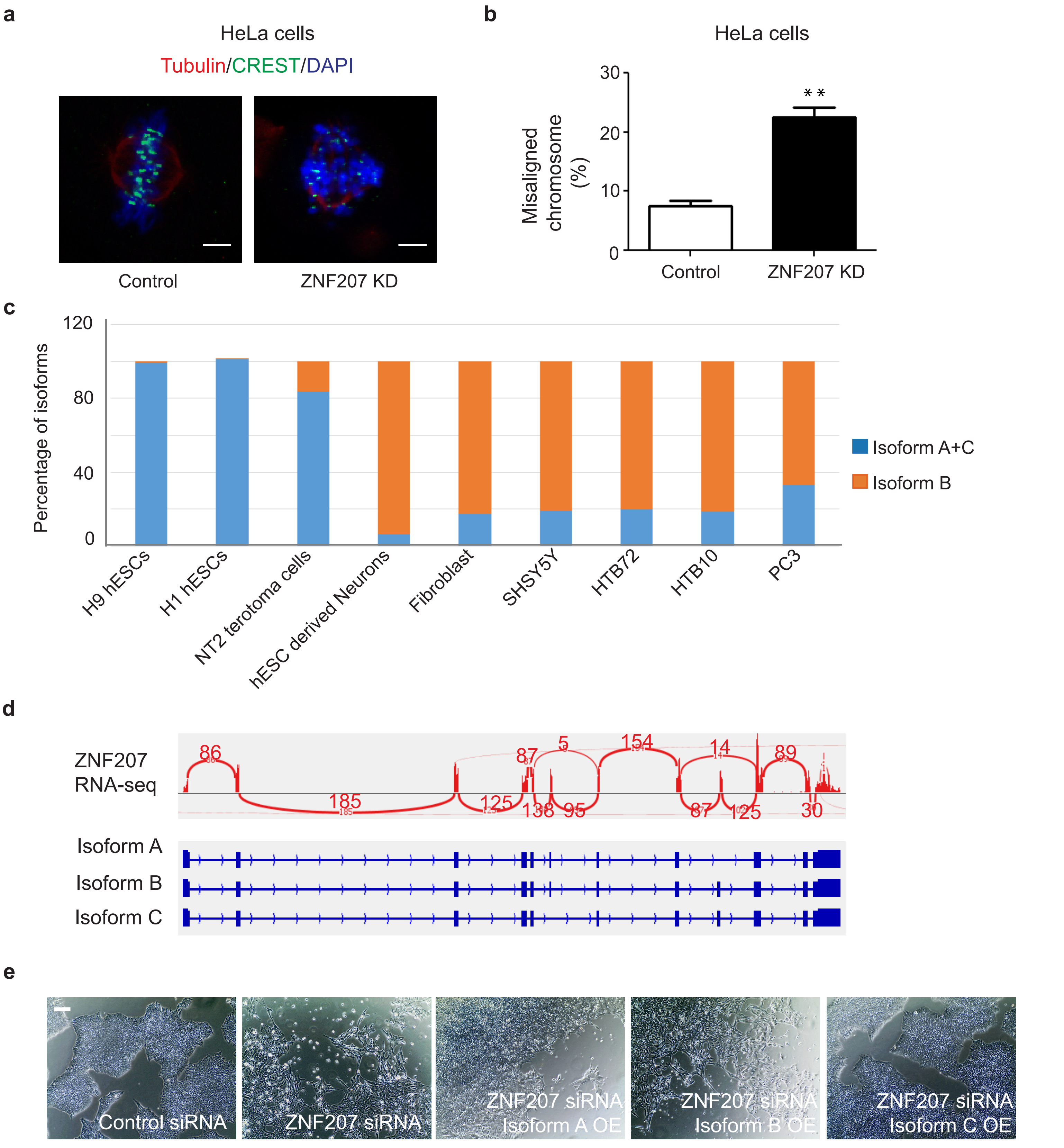

Supplementary Figure 7. Different isoforms are present in a variety of cell types and Isoform C is the major one that functions in HeLa cells. (**a**) Confocal images of mitotic cells with aligned chromosomes. ~100 mitotic cells were measured for each experiment and condition. Centromeres were detected by the CREST serum. Scale bar, 5μm. (**b**) Counts of mitotic cells with misaligned chromosomes in control and ZNF207 KD HeLa cells. **, p≤ 0.01. P-value was calculated by two-tailed Student's t-test. (**c**) RT-qPCR analysis to check the expression of isoforms in a variety of cell types. (**d**) Sashimi plot of ZNF207 gene in hESCs from RNA-seq data. The frequency of exon skipping is indicated by the numbers. (**e**) Bright field view of cells in different conditions. Scale bar, 50μm.

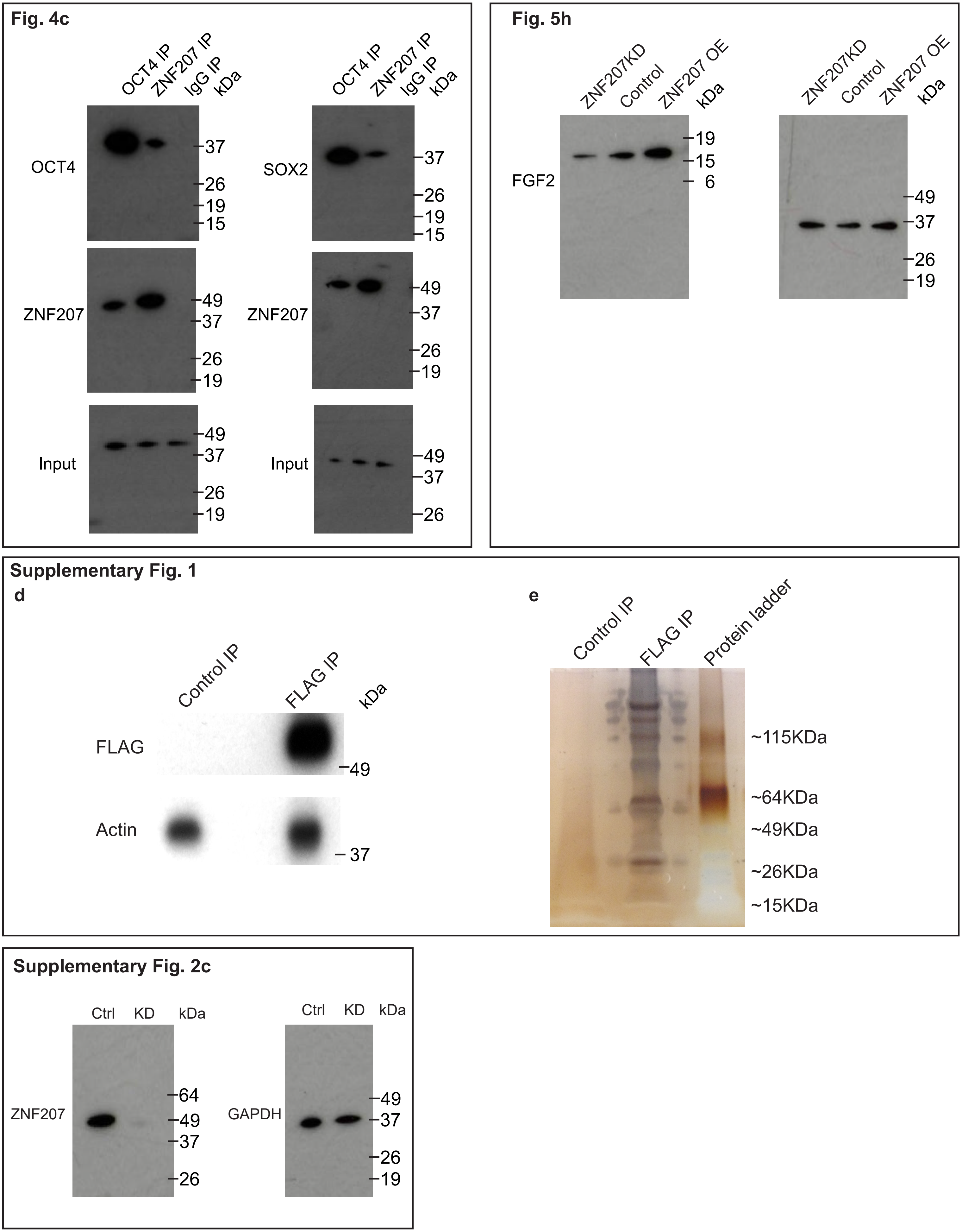

Supplementary Figure 8. Original blots for western blot analysis in this study.

Supplementary Table 1. Sequence and efficiency of Talen constructs targeting the proximal enhancer of *OCT4*

	Genomic Sequence	Talen RVD Sequence	Efficiency
Talen targeted site 1	TCCCCAGAGGGGGCAGCT ctaaccctaaacaagtgctc AACCCTTGAATGGGCCTGGA	HD HD HD HD NI NN NI NN NN NN NN NN HD NI NN HD NG	6%
		HD HD NI NN NN HD HD HD NI NG NG HD NI NI NN NN NN NG NG	
Talen targeted site 2	TGGCTCCCCTGGGGACTGCT tcctgctccccaaccccc AGTCCCAATCCCCTCACA	NN NN HD NG HD HD HD HD NG NN NN NN NN NI HD NG NN HD NG	1%
		NN NG NN NI NN NN NN NN NI NG NG NN NN NN NI HD NG	
Talen targeted site 3	TCCCCTGGGGACTGCTT cctgctccccaaccccc AGTCCCAATCCCCTCACA	HD HD HD HD NG NN NN NN NN NI HD NG NN HD NG NG	5%
		NN NG NN NI NN NN NN NN NI NG NG NN NN NN NI HD NG	
Talen targeted site 4	TGGGGACTGCTTCCTGCT ccccaacccccccagtccc AATCCCCTCACACAGA	NN HD NG HD HD HD HD NI NI HD HD HD HD HD HD NI NN NG	12%
		HD NG HD NG NN NI NI NN NN NN NN NI NG NG HD NG	

Supplementary Table 2. Summary of immunoprecipitated proteins with known binding to the proximal enhancer of OCT4 in hESCs

Gene	Description	Global FDR(False Discovery Rate)
**POU5F1**	POU class 5 homeobox1	<0.1%
**SOX2**	SRY (sex determining region Y)-box 2	<0.1%
**SALL4**	Sal-like 4 (Drosophila)	<0.1%
**HDAC2**	Histone deacetylase 2	<0.1%
**BRD4**	Bromodomain containing 4	<0.1%
**CTBP2**	C-terminal binding protein 2	<0.1%

Supplementary Table 3. Summary of the selected proteins for ChIP study

Gene	Description	Global FDR
**CBX**	Chromobox homolog 3	<0.1%
**HMGN1**	High mobility group nucleosome binding domain 1	<0.1%
**LIN28A**	Lin-28 homolog A (C. elegans)	<0.1%
**MBD3**	Methyl-CpG binding domain protein 3	<0.1%
**POU6F1**	POU class 6 homeobox 1	<0.1%
**SALL2**	Sal-like 2 (Drosophila)	<0.1%
**SMARCC1**	SWI/SNF related, matrix associated, actin dependent regulator of chromatin, subfamily c, member 1	<0.1%
**ZNF207**	Zinc finger protein 207	<0.1%
**ZNF706**	Zinc finger protein 706	<0.1%

Supplementary Table 4. DNA Sequence of codon-optimized ZNF207 overexpression construct

CACCATGGGCAGGAAGAAGAAGAAGCAGCTCAAGCCCTGGTGTTGGTACTGTAACCGAGATTTCGATGAC
GAGAAAATTTTGATCCAGCATCAGAAGGCAAAGCACTTTAAGTGCCATATCTGCCACAAGAAGCTCTATA
CAGGCCCTGGCCTCGCCATCCATTGCATGCAGGTGCACAAAGAGACTATCGATGCAGTACCTAATGCCAT
CCCCGGCCGAACAGACATTGAGCTGGAGATCTATGGTATGGAAGGAATACCTGAAAAAGACATGGACGAG
AGAAGACGCCTTTTGGAACAAAAAACACAGGAGTCTCAAAAGAAGAAACAGCAGGATGACTCCGATGAAT
ACGATGATGATGATTCAGCAGCTTCAACAAGCTTCCAGCCTCAGCCGGTACAGCCCCAACAGGGATATAT
TCCTCCAATGGCCCAGCCTGGACTTCCACCGGTTCCCGGAGCACCAGGGATGCCTCCAGGGATTCCACCT
CTGATGCCAGGAGTGCCTCCTCTCATGCCCGGTATGCCACCAGTGATGCCAGGCATGCCCCCAGGACTCC
ATCACCAGCGGAAATACACCCAATCTTTTTGTGGGGAGAATATTATGATGCCCATGGGAGGAATGATGCC
ACCTGGACCCGGGATTCCACCCCTGATGCCAGGCATGCCCCCCGGTATGCCACCCCCCGTGCCCCGCCCA
GGGATCCCTCCTATGACTCAAGCCCAGGCGGTGTCTGCCCCCGGCATCCTGAACCGCCCACCAGCCCCAA
CTGCAACTGTGCCCGCCCCCCAGCCCCCTGTTACCAAGCCGCTCTTTCCTTCTGCCGGCCAGATGGGAAC
TCCCGTAACATCCTCCAGCACCGCCTCAAGCAATAGTGAGTCTCTGTCCGCCAGTTCTAAGGCTCTCTTT
CCAAGTACCGCGCAGGCGCAGGCCGCAGTTCAGGGACCCGTGGGGACCGATTTTAAGCCCCTGAACTCCA
CCCCCGCTACTACTACTGAGCCTCCTAAGCCTACCTTCCCTGCATACACGCAGTCCACAGCTTCTACCAC
TAGCACCACAAACTCTACCGCCGCAAAGCCTGCCGCATCTATAACCTCCAAACCCGCCACTCTGACCACC
ACTAGTGCCACTAGTAAACTCATTCACCCAGACGAGGACATCAGCCTCGAGGAGAGACGAGCCCAGCTTC
CTAAATACCAACGGAATCTCCCTCGGCCTGGTCAGGCCCCGATTGGGAATCCACCTGTGGGACCCATTGG
TGGTATGATGCCCCCTCAGCCTGGGATTCCACAGCAACAAGGCATGCGGCCGCCAATGCCGCCCCACGGT
CAGTACGGCGGCCATCATCAAGGTATGCCAGGCTACCTCCCCGGCGCCATGCCCCCCTACGGGCAGGGCC
CCCCCATGGTGCCTCCATACCAAGGAGGCCCACCGAGGCCCCCCATGGGCATGCGGCCTCCGGTGATGTC
CCAGGGGGGCAGATATGACTATAAGGACCACGATGGTGATTACAAGGATCACGATATTGATTACAAGGAT
GACGACGACAAATGA

Supplementary Table 5. Sequence of ChIP primers

Primers	Direction	Sequences
1	Forward	CCACTAGCCTTGACCTCTGG
	Reverse	CAGTCTGGGCAACAAAGTGA
2	Forward	CTCCCACACCTCCATGTTCT
	Reverse	TGCCTAGGATTCTGGATGGA
3	Forward	GGGACCAGACTTTGGACTGA
	Reverse	CCTCCCAGGCTTCTTTGAAC
4	Forward	CTTCCTCTGGGGGACTCAG
	Reverse	CAAGCACTAGACCAGCAGCA
5	Forward	CGGTGGCTCATGCCTATAAT
	Reverse	TCGATCTCAGCTCACTGCAC

Supplementary Table 6. Sequence of siRNAs

Gene Name	Direction	Sequences	Accession #	Targeted Exons
MBD3	sense	CGGUGACCAAGAUUACCAAtt	NM_003926	3
	Antisense	UUGGUAAUCUUGGUCACCGgc		
SALL2	sense	CAGUGGAACCCAAGAAUAAtt	NM_005407	2
	Antisense	UUAUUCUUGGGUUCCACUGct		
LIN28A	sense	GAAGCGCAGAUCAAAAGGAtt	NM_024674	3
	Antisense	UCCUUUUGAUCUGCGCUUCtg		
POU6F1	sense	CCAAUUACCUGCUCAGAGAtt	NM_002702	ND
	Antisense	UCUCUGAGCAGGUAAUUGGga		
SOX2	sense	CCGUUGGUAAUUUAUAAUAgc	NM_003106	1
	Antisense	UAUUAUAAAUUACCAACGGtg		
OCT4	sense	GCAAAGCAGAAACCCUCGUgc	NM_001173531	3,4
	Antisense	ACGAGGGUUUCUGCUUUGCat		

Supplementary Table 7. Sequence for siRNA targeting *ZNF207*

Assay ID	Sequences	Interrogated Sequence	Targeted Exons	SiRNA Location
S15278	GAUGAAAGACGACGACUUC	NM_001032293.2	9	1178
S15279	CAAAUAGUACUGCAGCUAA	NM_001032293.2	3	437
S15280	CAUCAAGGCAUGCCAGGAU	NM_001032293.2	9	1090

Supplementary Table 8. Sequence of qPCR primers

Gene Name	Direction	Sequences
MBD3	Forward	GGCCACAGGGATGTCTTTTAC
	Reverse	GGCTCTTGTTCATCTTGCTC
SALL2	Forward	AGTTAATCTCGGACTGCGAAG
	Reverse	GAGGGTCAGTAGAACATGCG
LIN28A	Forward	CATGCAGAAGCGCAGATCAA
	Reverse	GGTGGCAGCTTGCATTCC
ZNF207	Forward	CCAGTCATTTTGCGGTGAAAAC
	Reverse	TGTGCTTGAGTCATTGGAGG
ZNF207 -iso a	Forward	GCATCATCAGAGAAAATACACCCAG
	Reverse	CATACCTGGTGGCATTCCAG
ZNF207 -iso b	Forward	GGACACCTGTCACAAGCTC
	Reverse	AACAGGTCCTTGGACAGCTG
ZNF207 -iso c-1	Forward	GAATGCCACCAGTTATGCCAG
	Reverse	AACAGGTCCTTGGACAGCTG
ZNF207 -iso c-2	Forward	CATACCTGGTGGCATTCCAG
	Reverse	AACAGGTCCTTGGACAGCTG
POU6F1	Forward	CTACAGCCAGTCAGCCATC
	Reverse	CCTCCCACAAACTCCATCAG
SOX2	Forward	CATGAAGGAGCACCCGGATTA
	Reverse	CGGGCAGCGTGTACTTATCC
OCT4	Forward	GGGGACCAGTGTCCTTTCC
	Reverse	GGGAAAGGGACCGAGGAGTA
GAPDH	Forward	ACACCATGGGGAAGGTGAAG
	Reverse	GTGACCAGGCGCCCAATA
GUSB	Forward	CATCGATGACATCACCGTCAC
	Reverse	ACAGGTTACTGCCCTTGACA

Supplementary Table 9. Antibodies used in this study

Primary/Secondary antibodies	Dilution	Company	Catalog #
ZNF207	ChIP: 5µg for 750µl chromatin pellet	Santa Cruz Biotechnology, Inc.	sc-271942
ZNF207	Western:1:2,000<br>IF: 1:500	Sigma	HPA017013
OCT4	Western: 1:1,000<br>IF: 1:500<br>ChIP: 5µg for 750µl chromatin pellet	Santa Cruz Biotechnology, Inc.	sc-8628
SOX2	Western: 1:1,000<br>IF: 1:200<br>IP: 1: 200	R&D Systems	MAB2018
FLAG	Western: 1:1,000<br>IP: 1: 200	Sigma	F7425
IgG	ChIP: 5µg for 750µl chromatin pellet	Santa Cruz Biotechnology, Inc.	Sc-66931
β-ACTIN	Western: 1:1,000	Cell Signaling	3700S
GAPDH	Western: 1:1,000	Proteintech	60004-1-lg
FGF2	Western: 1:1,000	Santa Cruz Biotechnology, Inc.	sc-79
NANOG	IF:1:200	Cell Signaling	#5232
TRA1-60	IF:1:200	Millipore	MAB4346
SOX17	IF:1:200	R&D Systems	AF1924
FOXA2	IF:1:500	Santa Cruz Biotechnology, Inc.	sc374375
NES	IF:1:500	Sigma	HPA026111
NCAM	IF:1:200	Stem Cell Technology	60021AD
OTX2	IF:1:500	R&D Systems	AF1979
α-TUBULIN	IF:1:500	Sigma	T9026
CREST	IF:1:500	Antibodies Incorporated	15-235
P55	IF:1:200	Santa Cruz Biotechnology, Inc.	Sc-13162
CBX	ChIP: 5µg for 750µl chromatin pellet	EMD Millipore	05-690
HMGN1	ChIP: 5µg for 750µl chromatin pellet	Bethyl Laboratories, Inc.	A302-363A
LIN28A	ChIP: 5µg for 750µl chromatin pellet	Proteintech	11724-1-AP
MBD3	ChIP: 5µg for 750µl chromatin pellet	Bethyl Laboratories, Inc.	A302-528A

POU6F1	ChIP: 5µg for 750µl chromatin pellet	Novus Biologicals	H00005463-D01
SALL2	ChIP: 5µg for 750µl chromatin pellet	Bethyl Laboratories, Inc.	A303-208A
SMARCC1	ChIP: 5µg for 750µl chromatin pellet	Cell Signaling Technology	11956S
ZNF706	ChIP: 5µg for 750µl chromatin pellet	Santa Cruz Biotechnology, Inc.	Sc-87771
488 Donkey Anti-Goat	IF:1:250	Jackson ImmunoResearch	705-545-147
594 Donkey Anti-Goat	IF:1:250	Jackson ImmunoResearch	705-585-147
488 Donkey Anti-Rabbit	IF:1:250	Jackson ImmunoResearch	711-545-152
594 Donkey Anti-Rabbit	IF:1:250	Jackson ImmunoResearch	711-585-152
488 Donkey Anti-Mouse	IF:1:250	Jackson ImmunoResearch	715-545-150
594 Donkey Anti-Mouse	IF:1:250	Jackson ImmunoResearch	715-585-150
