# Supplementary material for: A distinct isoform of ZNF207 controls self-renewal and pluripotency of human embryonic stem cells
Source: Nat Commun. 2018 Oct 22;9:4384. doi: 10.1038/s41467-018-06908-5 (PMC6197280; doi:10.1038/s41467-018-06908-5)
Supplement: Supplementary file 3 — Description of Additional Supplementary files [file 41467_2018_6908_MOESM3_ESM.pdf]

Supplementary Data file: Nuclear proteins identified by proteomics
