# Supplementary material for: A distinct isoform of ZNF207 controls self-renewal and pluripotency of human embryonic stem cells
Source: Nat Commun. 2018 Oct 22;9:4384. doi: 10.1038/s41467-018-06908-5 (PMC6197280; doi:10.1038/s41467-018-06908-5)
Supplement: Supplementary file 4 — Supplementary data file [file 41467_2018_6908_MOESM4_ESM.docx]

**Supplementary Data file: Nuclear proteins identified by proteomics**

| Gene Name | Description |
| --- | --- |
| ACIN1 | Apoptotic chromatin condensation inducer 1 |
| ACTN4 | Actinin, alpha 4 |
| AOF1 | Amine oxidase (flavin containing) domain 1 |
| APEX1 | APEX nuclease (multifunctional DNA repair enzyme) 1 |
| ARID1A | AT rich interactive domain 1A (SWI-like) |
| ASCC3L1 | Activating signal cointegrator 1 complex subunit 3-like 1 |
| BANF1 | Barrier to autointegration factor 1 |
| BOLA2 | BolA homolog 2 (E. coli) |
| BOLA2B | BolA homolog 2B (E. coli) |
| BRD4 | Bromodomain containing 4 |
| BUB3 | BUB3 budding uninhibited by benzimidazoles 3 homolog (yeast) |
| C14orf166 | Chromosome 14 open reading frame 166 |
| C19orf53 | Chromosome 19 open reading frame 53 |
| C20orf77 | Chromosome 20 open reading frame 77 |
| CACYBP | Calcyclin binding protein |
| CBX3 | Chromobox homolog 3 (HP1 gamma homolog, Drosophila) |
| CCNB1 | Cyclin B1 |
| CDC5L | CDC5 cell division cycle 5-like (S. pombe) |
| CIRBP | Cold inducible RNA binding protein |
| CLIC1 | Chloride intracellular channel 1 |
| CSRP2 | Cysteine and glycine-rich protein 2 |
| CTBP2 | C-terminal binding protein 2 |
| DDX42 | DEAD (Asp-Glu-Ala-Asp) box polypeptide 42 |
| DNAJC8 | DnaJ (Hsp40) homolog, subfamily C, member 8 |
| DPF2 | D4, zinc and double PHD fingers family 2 |
| DTD1 | D-tyrosyl-tRNA deacylase 1 homolog (S. cerevisiae) |
| EEF1B2 | Eukaryotic translation elongation factor 1 beta 2 |
| EEF2 | Eukaryotic translation elongation factor 2 |
| EIF4A3 | Eukaryotic translation initiation factor 4A, isoform 3 |
| EIF5A | Eukaryotic translation initiation factor 5A |
| EIF5AL1 | Eukaryotic translation initiation factor 5A-like 1 |
| ELAVL1 | ELAV (embryonic lethal, abnormal vision, Drosophila)-like 1 (Hu antigen R) |
| EMD | Emerin (Emery-Dreifuss muscular dystrophy) |
| ERH | Enhancer of rudimentary homolog (Drosophila) |
| FBL | Fibrillarin |
| FKBP3 | FK506 binding protein 3, 25kDa |
| GART | Phosphoribosylglycinamide formyltransferase, phosphoribosylglycinamide synthetase, phosphoribosylaminoimidazole synthetase |
| H2AFV | H2A histone family, member V |
| H3F3A | H3 histone, family 3A |
| H3F3B | H3 histone, family 3B (H3.3B) |
| HDAC2 | Histone deacetylase 2 |
| HMGA2 | High mobility group AT-hook 2 |
| HMGB2 | High-mobility group box 2 |
| HMGN1 | High-mobility group nucleosome binding domain 1 |
| HMGN2 | High-mobility group nucleosomal binding domain 2 |
| HMGN3 | High mobility group nucleosomal binding domain 3 |
| HMGN4 | High mobility group nucleosomal binding domain 4 |
| HN1L | Hematological and neurological expressed 1-like |
| HNRNPA2B1 | Heterogeneous nuclear ribonucleoprotein A2/B1 |
| HNRPA3 | Heterogeneous nuclear ribonucleoprotein A3 |
| HSPB1 | Heat shock 27kDa protein 1 |
| IGF2BP1 | Insulin-like growth factor 2 mRNA binding protein 1 |
| IGF2BP3 | Insulin-like growth factor 2 mRNA binding protein 3 |
| IK | IK cytokine, down-regulator of HLA II |
| ILF2 | Interleukin enhancer binding factor 2, 45kDa |
| JMJD1C | Jumonji domain containing 1C |
| KIAA0368 | KIAA0368 |
| LIN28 | Lin-28 homolog (C. elegans) |
| LRRC47 | Leucine rich repeat containing 47 |
| MAGED2 | Melanoma antigen family D, 2 |
| MAT2A | Methionine adenosyltransferase II, alpha |
| MBD3 | Methyl-CpG binding domain protein 3 |
| MCM2 | Minichromosome maintenance complex component 2 |
| MCM4 | Minichromosome maintenance complex component 4 |
| MCM5 | Minichromosome maintenance complex component 5 |
| MCM6 | Minichromosome maintenance complex component 6 |
| MCM7 | Minichromosome maintenance complex component 7 |
| MDN1 | MDN1, midasin homolog (yeast) |
| MKI67 | Antigen identified by monoclonal antibody Ki-67 |
| MSH2 | MutS homolog 2, colon cancer, nonpolyposis type 1 (E. coli) |
| MSH6 | MutS homolog 6 (E. coli) |
| NACA | Nascent polypeptide-associated complex alpha subunit |
| NAP1L1 | Nucleosome assembly protein 1-like 1 |
| NAT10 | N-acetyltransferase 10 |
| NCBP1 | Nuclear cap binding protein subunit 1, 80kDa |
| NIPBL | Nipped-B homolog (Drosophila) |
| NPM3 | Nucleophosmin/nucleoplasmin, 3 |
| NSBP1 | Nucleosomal binding protein 1 |
| NUDT21 | Nudix (nucleoside diphosphate linked moiety X)-type motif 21 |
| NUP153 | Nucleoporin 153kDa |
| NUP50 | Nucleoporin 50kDa |
| NUP93 | Nucleoporin 93kDa |
| PCBP2 | Poly(rC) binding protein 2 |
| PHF5A | PHD finger protein 5A |
| PHGDH | Phosphoglycerate dehydrogenase |
| PIN4 | Protein (peptidylprolyl cis/trans isomerase) NIMA-interacting, 4 (parvulin) |
| POLDIP3 | Polymerase (DNA-directed), delta interacting protein 3 |
| POU5F1 | POU class 5 homeobox 1 |
| POU6F1 | POU class 6 homeobox 1 |
| PPP1CA | Protein phosphatase 1, catalytic subunit, alpha isoform |
| PRPF31 | PRP31 pre-mRNA processing factor 31 homolog (S. cerevisiae) |
| PRPF40A | PRP40 pre-mRNA processing factor 40 homolog A (S. cerevisiae) |
| PRPF8 | PRP8 pre-mRNA processing factor 8 homolog (S. cerevisiae) |
| PSMA5 | Proteasome (prosome, macropain) subunit, alpha type, 5 |
| PSMA8 | Proteasome (prosome, macropain) subunit, alpha type, 8 |
| PSMB1 | Proteasome (prosome, macropain) subunit, beta type, 1 |
| PSMC1 | Proteasome (prosome, macropain) 26S subunit, ATPase, 1 |
| PTBP1 | Polypyrimidine tract binding protein 1 |
| RAN | RAN, member RAS oncogene family |
| RANGAP1 | Ran GTPase activating protein 1 |
| RBBP7 | Retinoblastoma binding protein 7 |
| RBM10 | RNA binding motif protein 10 |
| RBM3 | RNA binding motif (RNP1, RRM) protein 3 |
| RBMXL2 | RNA binding motif protein, X-linked-like 2 |
| RCC2 | Regulator of chromosome condensation 2 |
| RIF1 | RAP1 interacting factor homolog (yeast) |
| RPL3 | Ribosomal protein L3 |
| RPS19 | Ribosomal protein S19 |
| RRP9 | RRP9, small subunit (SSU) processome component, homolog (yeast) |
| SALL2 | Sal-like 2 (Drosophila) |
| SALL4 | Sal-like 4 (Drosophila) |
| SART3 | Squamous cell carcinoma antigen recognized by T cells 3 |
| SERF2 | Small EDRK-rich factor 2 |
| SF1 | Splicing factor 1 |
| SF3A1 | Splicing factor 3a, subunit 1, 120kDa |
| SF3A3 | Splicing factor 3a, subunit 3, 60kDa |
| SFRS3 | Splicing factor, arginine/serine-rich 3 |
| SFRS5 | Splicing factor, arginine/serine-rich 5 |
| SMARCA4 | SWI/SNF related, matrix associated, actin dependent regulator of chromatin, subfamily a, member 4 |
| SMARCB1 | SWI/SNF related, matrix associated, actin dependent regulator of chromatin, subfamily b, member 1 |
| SMARCC1 | SWI/SNF related, matrix associated, actin dependent regulator of chromatin, subfamily c, member 1 |
| SMARCD1 | SWI/SNF related, matrix associated, actin dependent regulator of chromatin, subfamily d, member 1 |
| SMC3 | Structural maintenance of chromosomes 3 |
| SMU1 | Smu-1 suppressor of mec-8 and unc-52 homolog (C. elegans) |
| SNRPA1 | Small nuclear ribonucleoprotein polypeptide A' |
| SOD1 | Superoxide dismutase 1, soluble (amyotrophic lateral sclerosis 1 (adult)) |
| SOX2 | SRY (sex determining region Y)-box 2 |
| SPIN1 | Spindlin 1 |
| SSB | Sjogren syndrome antigen B (autoantigen La) |
| SSBP1 | Single-stranded DNA binding protein 1 |
| SSRP1 | Structure specific recognition protein 1 |
| STIP1 | Stress-induced-phosphoprotein 1 (Hsp70/Hsp90-organizing protein) |
| SUB1 | SUB1 homolog (S. cerevisiae) |
| SUMO1 | SMT3 suppressor of mif two 3 homolog 1 (S. cerevisiae) |
| TCEA1 | Transcription elongation factor A (SII), 1 |
| TP53BP1 | Tumor protein p53 binding protein 1 |
| TPR | Translocated promoter region (to activated MET oncogene) |
| TPX2 | TPX2, microtubule-associated, homolog (Xenopus laevis) |
| TXN | Thioredoxin |
| U2AF2 | U2 small nuclear RNA auxiliary factor 2 |
| UBE2L3 | Ubiquitin-conjugating enzyme E2L 3 |
| WBP11 | WW domain binding protein 11 |
| WDHD1 | WD repeat and HMG-box DNA binding protein 1 |
| WIBG | Within bgcn homolog (Drosophila) |
| XPO1 | Exportin 1 (CRM1 homolog, yeast) |
| XRCC5 | X-ray repair complementing defective repair in Chinese hamster cells 5 (double-strand-break rejoining; Ku autoantigen, 80kDa) |
| XRCC6 | X-ray repair complementing defective repair in Chinese hamster cells 6 (Ku autoantigen, 70kDa) |
| XRN2 | 5'-3' exoribonuclease 2 |
| YBX1 | Y box binding protein 1 |
| YWHAE | Tyrosine 3-monooxygenase/tryptophan 5-monooxygenase activation protein, epsilon polypeptide |
| YWHAH | Tyrosine 3-monooxygenase/tryptophan 5-monooxygenase activation protein, eta polypeptide |
| ZNF207 | Zinc finger protein 207 |
